# Racial and ethnic disparities in genetic testing for hearing loss: a systematic review and synthesis

**DOI:** 10.1007/s00439-021-02335-7

**Published:** 2021-09-07

**Authors:** Stephanie L. Rouse, Michelle M. Florentine, Emily Taketa, Dylan K. Chan

**Affiliations:** 1 Department of Otolaryngology-Head and Neck Surgery, University of California-San Francisco, 2233 Post Street, Third Floor, San Francisco, CA 94115 USA; 2 Division of Pediatric Otolaryngology-Head and Neck Surgery, University of California-San Francisco, 2233 Post Street, Third Floor, San Francisco, CA 94115 USA; 3 Sackler School of Medicine, Tel Aviv University, Tel Aviv, Israel

## Abstract

Racial/ethnic disparities in the diagnostic efficacy of genetic testing for hearing loss have been described. These disparities may relate to differences in variant classification between different racial/ethnic groups, which may, in turn, derive from disparate representation of these groups in the published literature. We sought to quantify racial/ethnic disparities in the published literature on the human genetics of hearing loss. We conducted a search of PubMed for articles describing single-gene, multiple-gene, or whole-exome sequencing for individuals with sensorineural hearing loss. Data on the included subjects, including race/ethnicity and/or region of origin, a number of subjects tested, and method of testing, were extracted. 1355 populations representing 311,092 subjects from 1165 studies were included. Overall, subjects of European and Asian ancestry were equivalently represented, but those of Latino American, African, and indigenous North American ancestry were significantly underrepresented; over 96% of all subjects in the published literature were European or Asian. Within populations, the majority of subjects derived from a small subset of countries. The observed disparity was greater for multiple-gene and whole-exome sequencing than for single-gene sequencing. These findings illustrate the large disparity in the published literature on the genetics of hearing loss, and demonstrate the need for increased representation of Latino American, African, and indigenous North American populations.

## Introduction

Sensorineural hearing loss (SNHL) is the most common congenital sensory disorder, affecting 1 in 500 newborns, and over 80% of those 85 years of age and older (Morton and Nance 2006; Fortnum et al. 2001). Identifying a genetic etiology of hearing loss provides valuable prognostic information, allows early detection of syndromic forms of SNHL prior to overt syndromic phenotypes, and facilitates time-sensitive counseling (Kimberling et al. 2010; Brodie et al. 2020; Shearer et al. 2019). Establishing etiologic diagnoses for deafness, however, is challenging due to the multitude of potential causes, clinical variability, phenotypic overlap and genetic heterogeneity of hearing loss (Hilgert et al. 2009). The advance of next-generation sequencing (NGS) technologies has made genetic testing cost-effective, increasing the availability of testing and transforming clinical diagnostic practice. Comprehensive genetic testing (CGT) has rapidly become a valuable tool for the identification of a genetic cause of hearing loss in deaf and hard-of-hearing (D/HH) patients (Pandya 2016). As genetic testing expands, it is important to ensure it is conducted equitably.

There is growing acknowledgement of disparities in genetic testing in hearing loss based on race and ethnicity. Multiple groups have found that the rate of molecular diagnosis as well as the spectrum of genes implicated by CGT for SNHL varies widely by racial/ethnic group, with Asians and Whites having higher diagnostic rates compared with Blacks and Hispanics (Sloan-Heggen et al. 2016; Yan et al. 2016; Florentine et al. 2021) While it is possible that true differences exist between these groups in the incidence of genetic hearing loss, it is also possible that the decreased diagnostic efficacy of genetic testing among Blacks and Hispanics derives from the relative paucity of knowledge on the genetics of hearing loss in these groups, both in terms of undiscovered deafness genes and uncertain interpretation of variants found in known hearing-loss genes. Indeed, *GJB2*, the most frequently studied gene in hearing-loss genetics, is commonly affected in Whites and Asians, but rarely in Blacks (Chan and Chang 2014; Lebeko et al. 2015).

Because interpretation of sequence variants is a crucial element of accurate genetic diagnosis and discrepancies in variant interpretation can have serious, harmful implications for patient care, standards designated by the American College of Medical Genetics for classification of variants require substantial evidence to categorize a variant as disease-causing (Amendola et al. 2016; Booth 2018; Harrison et al. 2016). Therefore, underrepresentation of groups in genetic studies, and the resultant underrepresentation in the knowledge base upon which variant classification is performed, may constrain diagnostic power for these groups. Indeed, the proportion of known deafness-causing variants is greater in Whites and Asians, whereas the rate of variants of unknown significance (VUSs) is high among people of African or Central American descent (Yan et al. 2016; Florentine et al. 2021). Conversely, targeted ascertainment of specific populations can have deleterious effects on knowledge and clinical management for both targeted and non-targeted populations (Carmeli 2004). Thus, these racial/ethnic disparities in genetic testing for hearing loss must be addressed to achieve the ethical principles of non-maleficence and justice: non-maleficence through avoidance of harmful management decisions made from incomplete genetic knowledge, and justice through equitable representation and treatment of children across racial/ethnic groups.

In this study, we sought to quantify the racial/ethnic disparity in the published literature on the genetics of hearing loss. In doing so, we aim to describe the extent to which racial/ethnic groups may be underrepresented in the hearing-loss genetic literature, which may underlie disparities in the diagnostic efficacy of genetic testing for hearing loss and our understanding of deafness across all populations. Understanding how the efficacy and limitations of genetic testing are affected by racial and ethnic disparities is necessary for health equity.

## Methods

### Systematic review

We performed a systematic review in accordance with PRISMA guidelines (Moher et al. 2009). We conducted three searches to compile the studies included in this systematic review. First, we searched PubMed on March 30, 2021 using the search parameter “COL11A1 OR MYO7A OR MYH14 OR USH2A OR OTOG OR TNC OR MCM2 OR MYH9 OR CDH23 OR COL11A2 OR TCOF1 OR TECTA OR PCDH15 OR SCL26A4 OR STRC OR TJP2 OR KCNQ4 OR OTOF OR TMC1 OR ALMS1 OR COCH OR DFNA5 OR DFNB59 OR EYA1 OR MYO15A OR PAX3 OR PDZD7 OR CHD7 OR COL4A3 OR DIAPH3 OR EYA4 OR FGFR3 OR MITF OR MYO6 OR OTOGL OR POU4F3 OR SEMA3E OR SLC17A8 OR SOX10 OR TMPRSS3 OR ("genetic testing") OR (exome) AND (hearing OR deafness) NOT (review) NOT (screening)” with the “human only” filter. This search identified studies in which either WES, multiple-gene testing (including CGT), or single-gene testing for common genes involved in hearing loss, excluding *GJB2*, was performed. We assembled this list of individual genes by examining a database of pediatric patients with SNHL who underwent CGT at UCSF (Florentine et al. 2021). Each of the 41 genes included was possibly causative for hearing loss in two or more patients within a racially and ethnically diverse cohort. This search identified 1635 articles.

We performed two separate searches on *GJB2*, given the large number of publications related to this gene and the existence of a prior large systematic review (Chan and Chang 2014). First, we extracted information on GJB2 from a database compiled by the corresponding author (DKC) on July 18, 2012 with the search criteria “(GJB2 OR Connexin 26 OR Cx26) AND (hearing OR deafness),” which included studies through 2012. We re-reviewed the 245 articles evaluated for this systematic review according to the inclusion and exclusion criteria for this study, and extracted data as described below. To additionally identify studies published during or after 2013 while avoiding duplicates from the global search, we performed a second, separate search for *GJB2* on April 15, 2021 using these parameters: “(GJB2) AND (hearing or deafness) NOT (screening OR review OR COL11A1 OR MYO7A OR MYH14 OR USH2A OR OTOG OR TNC OR MCM2 OR MYH9 OR CDH23 OR COL11A2 OR TCOF1 OR TECTA OR PCDH15 OR SCL26A4 OR STRC OR TJP2 OR KCNQ4 OR OTOF OR TMC1 OR ALMS1 OR COCH OR DFNA5 OR DFNB59 OR EYA1 OR MYO15A OR PAX3 OR PDZD7 OR CHD7 OR COL4A3 OR DIAPH3 OR EYA4 OR FGFR3 OR MITF OR MYO6 OR OTOGL OR POU4F3 OR SEMA3E OR SLC17A8 OR SOX10 OR TMPRSS3 OR (“genetic testing”) OR (exome))” with the “human only” filter. This search identified 165 articles.

We included studies based on title, abstract, and full-length paper (when necessary) according to these criteria:

- Inclusion criteria:
  i. Primary report of human subjects with hearing loss
  ii. Single-gene, multiple-gene, or whole-exome sequencing was performed.
  iii. Geographic ancestry, race, or ethnicity of subjects, region where the study was performed, or the country of the corresponding author was identifiable.
- Exclusion criteria:
  i. Review or screening studies
  ii. Studies in which specific variants were studied rather than complete gene sequencing performed

### Search results

From these three searches, we identified a total of 2,045 studies, of which 1,165 were included, after applying inclusion/exclusion criteria and eliminating duplicate entries. From these studies, we collected data including article identifiers (author, year, PMID) and, for each distinct, identifiable population within the study, geographic ancestry of subjects if stated, the race/ethnicity if stated, region of subjects inferred from the region where the study was performed, country of corresponding author, genetic testing method (multiple gene testing, exome sequencing, or single-gene testing), as well as the sample size of those who underwent genetic testing, including controls. Three reviewers each independently reviewed one-third of the 1,635 articles from the broad search results. A fourth reviewer independently reviewed 410 articles from the two *GJB2* search results. When questions arose about study inclusion or data extraction, at least one additional author reviewed the study and a decision was made after discussion. Less than 5% of articles were brought up for review and discussion by multiple authors. We extracted data from abstracts when available and from the full-text review when necessary. We did not explicitly assess the risk of bias for each individual article given the large numbers of studies and case study/case series design for all.

### Population categorization

The primary outcome measure in this systematic review and synthesis is the geographic ancestry of populations described within included studies. We reviewed each study to identify unique populations, each defined by the following attributes:

1. Geographic ancestry—region of origin of the population (country, when available, and/or region as defined by United Nations Geoscheme)
2. Racial/ethnic group of the population
3. Country of the corresponding author
4. Number of subjects in the population
5. Type of genetic testing performed.

We classified subjects as being of European, Asian, African, or American descent, with specific country assigned when available. When available, we used geographic ancestry or race/ethnicity to assign populations. If neither were available, we assigned the population to the region where the study took place (for example, a population described in a study from China without race, ethnic, or country of ancestral origin explicitly stated would be classified as Chinese). For each population, we recorded whether a population had an explicitly defined geographic ancestry, race, or ethnicity, or whether ancestry was inferred from the study site. We then assigned the defined unique region of origin to a population as described below.

1. European ancestry:
  - Region of ancestral origin within Europe;
  - Race/Ethnicity described as “Caucasian” or “White”;
  - Region assigned to the population was located in Europe, United States, Canada, Australia, or New Zealand; or
  - Participants whose race/ethnicity was stated as Ashkenazi Jewish
2. African ancestry:
  - Region of ancestral origin within Africa
  - Race/Ethnicity described as “Black” or “African American”; or
  - Region assigned to the population was located in Africa
3. Asian ancestry:
  - Region of ancestral origin within Asia;
  - Race/Ethnicity described as “Asian”; or
  - Region assigned to the population was located in Asia, including the Middle East (i.e. Western Asia, as defined by the United Nations Geoscheme).
4. Latino American ancestry:
  - Region of ancestral origin within Central or South America;
  - Race/Ethnicity described as “Hispanic,” or “Latino”; or
  - Region assigned to the population was located in South or Central America.
5. North American ancestry:
  - Region of ancestral origin within North America; or
  - Race/Ethnicity described as “Native American” or “Native Hawaiian.”
6. Subjects of defined mixed ancestry/race/ethnicity were assigned once to each population to reflect their contribution to the literature for each group.

We recorded the number of individual subjects comprising each population. In cases where sample size information was limited to the number of families rather than individual participants, we considered each family as a sample size of one. Finally, we only included studies if, at a minimum, sequence analysis of a single gene was performed. We recorded the broad type of genetic testing performed for each population as single-gene testing, multiple-gene testing, or whole-exome sequencing.

### Statistical analysis and human subjects protection

We used Stata/MP software version 16 (College Station, Texas) running on Microsoft Remote Desktop (Windows) for descriptive analysis. The retrospective review to identify the 41 common hearing-loss genes to be included in the search was approved by the Institutional Review Board of UCSF. We determined the literature review to be exempt from IRB review.

## Results

### Systematic review

**Fig. 1:**
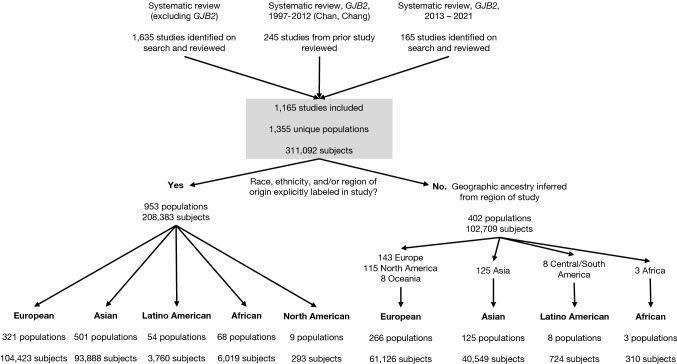
Flowchart of a systematic review and population assignment

**Fig. 2:**
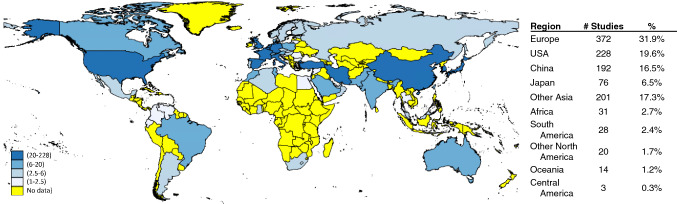
Home country of the corresponding author. The number of studies with a corresponding author from each country is indicated. Darker blue indicates more studies (range as indicated in legend); yellow indicates no studies originating from that country

The three search criteria identified 2045 articles, of which 1165 met the criteria for inclusion and data synthesis (Fig. 1). These studies encompassed 1355 populations and 311,092 subjects who underwent genetic testing across 87 countries and 6 continents from 1992 to 2021. The majority of studies were performed in the United States (20%), Europe (32%), China (16%), and Japan (7%), with relatively few studies originating from Latin America and Africa (Fig. 2).

### Population distribution

**Fig. 3:**
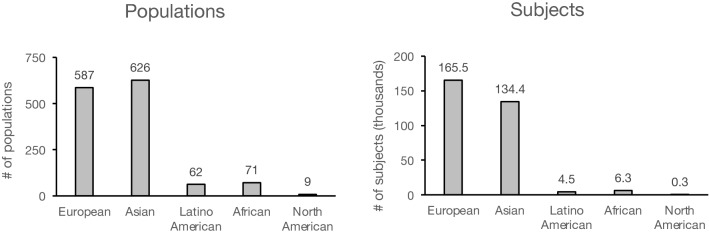
Racial/ethnic distribution of published hearing-loss genetic testing data. The number of populations (left) and subjects (right) attributable to the indicated geographic ancestral groups is described for 1165 included studies, comprising 1355 populations and 311,092 subjects

We sought to identify the population makeup of published literature on the genetics of hearing loss. Race, ethnicity, and/or geographic region of origin of subjects was explicitly stated for 953 (70%) of studies and inferred based on the region where the study was performed for the remaining 402 (30%) studies. Of the 1,355 populations, 43% were European, 46% were Asian, 5% were African, 6% were Latino American, and 0.7% were indigenous North American (Fig. 3, left). Of the 311,092 subjects included, 53% were European, 43% Asian, 2% African, 1% Latino American, and 0.1% North American (Fig. 3, right). These findings demonstrate that, compared with European populations, Asian populations are represented roughly equivalently, but Latino American populations are underrepresented in genetic testing literature by a factor of 9.5 to 1, African populations by 8.3 to 1, and indigenous North American populations by 65 to 1. European subjects outnumber Latino American subjects by 37:1, Africans by 26:1, and indigenous North Americans by 565:1. Overall, Europeans and Asians comprise 96.4% of all reported subjects in publications on genetic testing for hearing loss.

### Geographic distribution

**Fig. 4:**
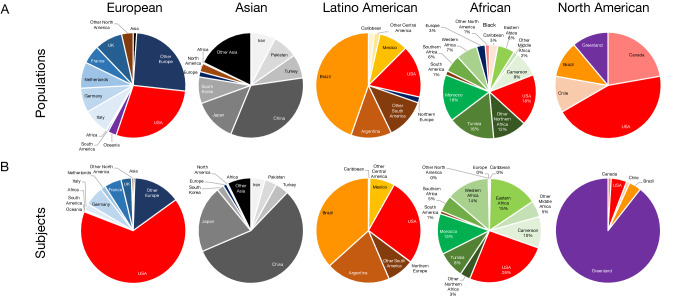
Countries/regions of origin for populations (**A**) and subjects (**B**) contributing to each geographic ancestral group

Within continental populations, the distribution of countries for the included populations introduces yet another element of bias in the representation within each group. We describe the distribution of countries/regions of origin within the continental groups for both populations and subjects (Fig. 4). For this geographic analysis, we did not include 34 studies that did not explicitly state a single specific country of origin for the included populations.

The majority of subjects of European ancestry originated from the United States (66%) or Europe (33%), and the majority of Asian subjects were from East (80%) and Western (10%) Asia, especially China (56%) and Japan (21%), with limited representation from Southern (7%) and Southeastern (1%) Asia. The majority of Latino American participants originated from Brazil (37%) and the United States (27%), with scant contribution from Central America (8%) or other parts of South America outside of Brazil and Argentina. Finally, the majority of African participants originated from the United States (26%) and Northern Africa (24%), including Tunisia and Morocco, despite the majority of the African population living outside these regions, particularly sub-Saharan Africa. Finally, there were extremely few studies on Native American, Alaskan, Hawaiian, and Pacific Islander populations, with the majority of subjects reported from a single study from Greenland (89%).

### Studies over time

**Fig. 5:**
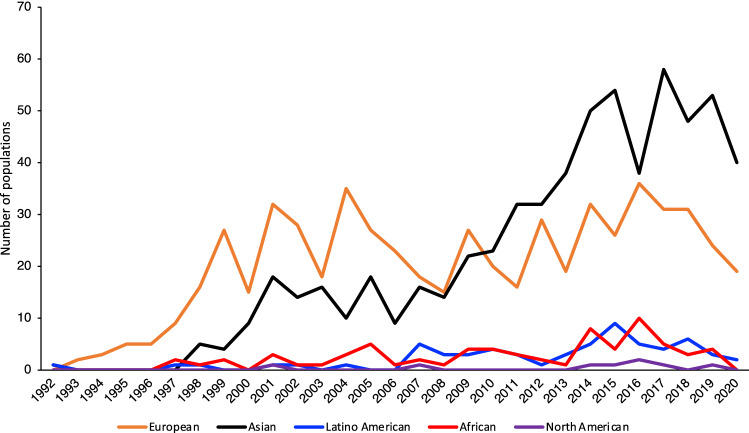
Representation of geographic ancestral groups in published studies over time

We analyzed the number of populations described for each geographic ancestral group over time to understand trends in studied racial/ethnic groups between 1992–2020. On average, 20 studies have been published per year on European populations, 21 on Asians, 2 on Latino Americans, 2 on Africans, and 0.3 on indigenous North Americans, with Asian studies overtaking European studies since 2010 (Fig. 5).

### Genetic testing method

**Fig. 6:**
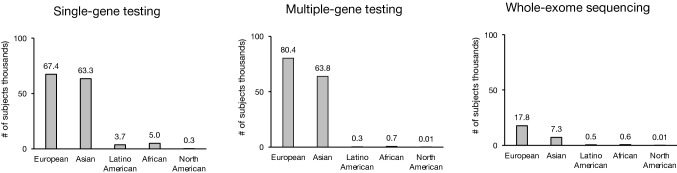
Representation of subjects from different geographic ancestral groups in published studies by genetic testing type

We categorized the genetic testing method for each population as single-gene testing (45% of all subjects), multiple-gene testing (47%), or whole-exome sequencing (8%). While European and Asian subjects were roughly equally represented in all three testing modalities, the magnitude of the disparity between White and African, Latino American, and North American subjects varied significantly by testing type. Whereas European subjects outnumbered Latino American and African subjects in single-gene testing reports by 18- and 13-fold, respectively, they outnumbered Latino American and African subjects in multiple-gene testing by 250- and 113-fold, and whole-exome sequencing by 39- and 29-fold (Fig. 6).

## Discussion

Recent advances in technology, decrease in expense, and broadening of insurance coverage has made NGS more accessible and ubiquitously used. While CGT is one of the strongest tools in the clinical evaluation of SNHL, it must be equivalently accessible and informative across all populations, lest it exacerbate existing disparities. Several studies have found that Hispanic and Black populations are currently less served by genetic testing, with higher numbers of VUSs and poorer diagnostic rates (Yan et al. 2016; Florentine et al. 2021). In an attempt to understand and address the source of this disparity in genetic testing, we reviewed the distribution of studies on genetic testing in hearing loss.

In a systematic review of 1165 studies describing 1355 unique populations and 311,092 subjects, we found striking differences in the number of studies, participants, and populations based on racial/ethnic group. European and Asian populations were represented 10 times more than Latino American or African populations, and over 70 times more than indigenous North Americans. When considering the number of individual subjects, the disparity was even larger: compared with European subjects, there were 37-fold fewer Latino American, 26-fold fewer African, and 565-fold fewer indigenous North Americans. Even within broad racial/ethnic groups, the distribution of countries from which these populations were derived is uneven; for example, 57% of subjects in the Latino American category are from Brazil and Argentina, and 77% of subjects in the Asian category are from China and Japan, suggesting that representation of other sub-groups is limited. Studies overall originated predominantly from a small set of countries in Europe, North America, and East Asia, with over 82% of corresponding authors from these regions. Though there were occasional examples of collaborative work between groups from these regions and other, less-studied parts of the world, further development of such collaborations may help improve representation. Even within a region, the promotion of community engagement and participatory research in underrepresented minority communities can significantly improve inequity and address disparities. Whether the increased representation is achieved by collaboration with populations in underserved countries or minority communities in otherwise well-represented ones, meaningful and ethical engagement of the target populations is critical.

While trends over time suggest a slow increase in representation of African and Latino American populations, and Asian populations overtaking Europeans in the literature, the gap remains large. Furthermore, compared with single-gene testing, more advanced genetic testing strategies, such as multiple-gene testing and whole-exome sequencing, which carry greater potential for genetic discovery, are subject to far greater disparities, with Europeans and Asians comprising nearly 96% of all subjects who underwent whole-exome sequencing and over 99% of all subjects who underwent multiple-gene testing, including CGT. Expansion of more advanced genetic testing technology in intentional ways may offer an opportunity to close the gap in understanding normal variation, identifying pathogenic variants, and discovering new deafness genes in underrepresented populations.

The racial/ethnic disparity in the published literature on hearing-loss genetics is stark. Though African and Latino American people make up 26% of global populations, they comprise only 3% of subjects in hearing-loss genetics studies. In contrast, European individuals comprise 15% of the global populations and 53% of reported subjects. This underrepresentation in the literature likely underlies the previously reported poor diagnostic efficacy of genetic testing for hearing loss in sub-Saharan African (4%) and Guatemalan (0%) probands, compared to a 28% diagnostic rate for all probands from non-sub-Saharan African countries (Yan et al. 2016). These findings accord with previous reports on genetic testing in general (Suther and Kiros 2009); a 2009 analysis revealed 96% of participants in genome-wide association studies (GWAS) were of European descent. A 2016 update showed that White participants continued to make up 81% of all samples in GWAS studies while comprising only 16% of the global population; in comparison, Black and Hispanic subjects comprised only 2% and < 0.5%, respectively (Martin et al. 2019). Indigenous American and Pacific Islander populations experience particular challenges in accessing testing; we found that Native American and Native Hawaiian groups are barely represented at all in the literature, comprising a mere 0.01% of all subjects reported (D’Angelo et al. 2020).

This trend in racial/ethnic disparities has been well described in genetic testing for other diseases. Broadly, patients of African and Asian ancestry are more likely than those of European ancestry to receive ambiguous genetic test results after exome sequencing or be told that they have VUSs (Petrovski and Goldstein 2016). Cardiomyopathy suffers from similar diagnostic inequity, in which genetic testing is more likely informative for those with well-characterized variants predominantly from European populations, as current standards prioritize limiting false-positive rates over test sensitivity (Walsh et al. 2019; Ho et al. 2018). As a result, patients of African ancestry are more likely than those of European ancestry to be falsely told their variant increases their risk of developing life-threatening hypertrophic cardiomyopathy (Gerhard et al. 2018; Manrai et al. 2016). These analyses illustrate how unequal representation of genetic variation can negatively affect present genomic interpretation in individuals of non-European ancestry (Petrovski and Goldstein 2020). In contrast with studies in other medical conditions, Asians are well represented in the hearing-loss genetics literature, with 46% of populations of Asian origin and the number of studies conducted in Asian populations surpassing the number of studies in European populations in 2010. The majority of this representation, however, is from East Asia, and China in particular; representation from other Asian regions, especially Southeast Asia, is still very poor.

This study has several limitations. First, there was considerable heterogeneity in descriptions of race, ethnicity, and ancestry. Many of the studies included in this analysis made no explicit mention of the genetic ancestral, racial, or ethnic background of populations, and if they did, they did not specify whether the categorization was self-identified by patients/populations, inferred by the study investigators, or determined based on genetic ancestry analysis. Thus, we made multiple assumptions to assign a population to groups defined by race/ethnicity or region of residence. These assignments can lack complexity, underrepresent certain groups, and may not align with how these populations identify themselves. In particular, subjects of Latino American ancestry primarily comprised self-identified Hispanic individuals in the United States and subjects from studies performed in Central and South America. We did this to try to measure the representation of indigenous American ancestry in the literature. However, genetic admixture analysis demonstrates that Latino populations contain significant European ancestry (González Burchard et al. 2005). Thus, this study has likely underestimated the true representation and disparity of indigenous American ancestry in the hearing-loss genetic literature. Future studies should be more rigorous in defining populations based on genetic ancestry and must be precise in describing how race, ethnicity, and genetic ancestry are determined and reported (Borrell et al. 2021). For example, using techniques such as genetic admixture analysis and definitions consistent with established standards such as the 1000 Genomes Project can make findings more precise and facilitate data sharing (IGSR 2021).

The structure of our search and method of data extraction from articles had limitations. Search terms, while intended to generate a thorough and representative sampling of studies pertaining to genetic testing and hearing loss, were not necessarily comprehensive. Additionally, we introduced a potential source of bias as we only queried one database (PubMed). While we attempted to address systemic bias by performing comparative analysis of studies and populations against each other, we did not explicitly address bias on an article-by-article basis. As all articles were case studies or case series, only 2 of 7 domains (Selective Reporting and Complete Data) are potentially relevant from Cochrane guidelines, and our goal of comparative analysis of studies and their respective study populations against each other mitigates the impact of these potential biases on our analysis (Cochrane Handbook for Systematic Reviews of Interventions 2021). We further mitigated systematic bias by including non-English studies. Data extraction was systematic, but imperfect given incomplete data. Given a large number of articles, most were reviewed by only one author, which may increase the error rate. Despite these methodological limitations, none are expected to systemically bias the comparisons or the conclusions, particularly given the large magnitude of the effects seen.

Consideration of race/ethnicity in scientific research and medicine is complex. This study does not address issues of race/ethnicity as a social construct or a biological construct relating to genetic ancestry but merely is intended to illustrate the disparities in the literature and underrepresentation of broadly defined groups (Burchard et al. 2003; Cooper et al. 2003). While there may be conflicting perspectives on the inclusion of race as a factor in the study of medicine and science, more information on underrepresented groups, as defined by genetic ancestry, country of origin, or self-identification, is clearly needed. Patient populations represented in studies directly inform clinical interpretation, decision making, and outcomes. Advances in screening and increases in availability have the potential to help ameliorate disparities present in hearing loss, but for that to be a reality, genetic testing must be equally informative for all groups (Bush et al. 2017). If applications of new technology, like NGS, continue to be applied disproportionally in overrepresented populations, the models generated from newly available data risk perpetuating and exacerbating health disparities (Popejoy and Fullerton 2016).

Finally, while it is commonplace to compare the percent of subjects studied to the percent of the global population belonging to a particular group, even distribution of studies along these population proportions is likely not the best approach for allocation of future research. These subgroups do not represent homogenous populations, and genetic diversity within single populations is often larger than between groups. For example, while our findings suggest that Asians are well represented in the literature, and therefore do not need to be specifically targeted in future research, the “Asian” group aggregates many divergent populations into one group that is very heterogeneous. 77% of studies of Asian populations only included Chinese and Japanese participants; Cambodian and Hmong immigrants have poorer overall health outcomes than other Asian Americans and are not represented by these works (Srinivasan et al. 2015). Additionally, several studies indicate greater genetic diversity between individuals of the same race than between individuals of different races (Baye 2011; Lewontin 1972; Kaessmann et al. 2001). This has been especially noted among individuals of African ancestry (Mersha and Abebe 2015). It is estimated that because of shorter linkage disequilibrium, a GWAS of the African population would require approximately 1.5 million SNPs to achieve the same resolution as a study of a European population using 0.6 million SNPs (Jallow et al. 2009). Therefore, allocating research to European and African populations proportional to their populations would underserve the genetic diversity of African populations.

## Conclusion

This systematic review and synthesis demonstrate a wide disparity in the published literature on the genetics of hearing loss, with Latino American, African, and indigenous North American populations vastly underrepresented compared to Europeans and Asians. Increasing the representation of these populations in future clinical testing and research efforts is necessary to improve the efficacy and equity of genetic testing for hearing loss.

## Data Availability

All relevant data are included in this manuscript.
